# Identification, Shiga toxin subtypes and prevalence of minor serogroups of Shiga toxin-producing *Escherichia coli* in feedlot cattle feces

**DOI:** 10.1038/s41598-021-87544-w

**Published:** 2021-04-21

**Authors:** Kaylen M. Capps, Justin B. Ludwig, Pragathi B. Shridhar, Xiaorong Shi, Elisabeth Roberts, Chitrita DebRoy, Natalia Cernicchiaro, Randall K. Phebus, Jianfa Bai, T. G. Nagaraja

**Affiliations:** 1 Department of Diagnostic Medicine/Pathobiology, Kansas State University, Manhattan, KS 66506 USA; 2 E. Coli Reference Center, Department of Veterinary and Biomedical Sciences, The Pennsylvania State University, University Park, PA 16802 USA; 3 Department of Animal Sciences and Industry/Food Science Institute, Kansas State University, Manhattan, KS USA; 4 Veterinary Diagnostic Laboratory, Kansas State University, Manhattan, KS 66506 USA

**Keywords:** Microbiology, Bacteria, Infectious-disease epidemiology

## Abstract

Shiga toxin-producing *Escherichia coli* (STEC) are foodborne pathogens that cause illnesses in humans ranging from mild to hemorrhagic enteritis with complications of hemolytic uremic syndrome and even death. Cattle are a major reservoir of STEC, which reside in the hindgut and are shed in the feces, a major source of food and water contaminations. Seven serogroups, O26, O45, O103, O111, O121, O145 and O157, called ‘top-7’, are responsible for the majority of human STEC infections in North America. Additionally, 151 serogroups of *E. coli* are known to carry Shiga toxin genes (*stx*). Not much is known about fecal shedding and prevalence and virulence potential of STEC other than the top-7. Our primary objectives were to identify serogroups of STEC strains, other than the top-7, isolated from cattle feces and subtype *stx* genes to assess their virulence potential. Additional objective was to develop and validate a novel multiplex PCR assay to detect and determine prevalence of six serogroups, O2, O74, O109, O131, O168, and O171, in cattle feces. A total of 351 strains, positive for *stx* gene and negative for the top-7 serogroups, isolated from feedlot cattle feces were used in the study. Of the 351 strains, 291 belonged to 16 serogroups and 60 could not be serogrouped. Among the 351 strains, 63 (17.9%) carried *stx*1 gene and 300 (82.1%) carried *stx*2, including 12 strains positive for both. The majority of the *stx*1 and *stx*2 were of *stx*1a (47/63; 74.6%) and *stx*2a subtypes (234/300; 78%), respectively, which are often associated with human infections. A novel multiplex PCR assay developed and validated to detect six serogroups, O2, O74, O109, O131, O168, and O171, which accounted for 86.9% of the STEC strains identified, was utilized to determine their prevalence in fecal samples (n = 576) collected from a commercial feedlot. Four serogroups, O2, O109, O168, and O171 were identified as the dominant serogroups prevalent in cattle feces. In conclusion, cattle shed in the feces a number of STEC serogroups, other than the top-7, and the majority of the strains isolated possessed *stx*2, particularly of the subtype 2a, suggesting their potential risk to cause human infections.

## Introduction

Shiga toxin-producing *Escherichia coli* (STEC) are major foodborne pathogens responsible for human illnesses ranging from mild enteritis to hemorrhagic colitis, which in a few cases lead to complications, including hemolytic uremic syndrome (HUS) and even death^1–3^. The primary virulence factors of STEC are two types of Shiga toxins, Stx1 and Stx2, encoded by *stx*1 and *stx*2, which are carried on a lambdoid prophage^4^. The two toxins have similar modes of action but differ immunologically. Based on differences in nucleotide sequences of *stx* genes, amino acid sequences of Stx proteins and the degree of cytotoxicity, Shiga toxins and Shiga toxin genes are classified into several variants or subtypes^5, 6^. The *stx*1 gene has four subtypes, *stx*1a, *stx*1c, *stx*1d, and *stx*1e and *stx*2 has 12 subtypes, *stx*2a, *stx*2b, *stx*2c, *stx*2d, *stx*2e, *stx*2f., *stx*2g, *stx*2h, *stx*2i, *stx*2j, *stx*2k, and *stx*2l^4, 7, 8^. The severity and complications of human illnesses are influenced by Shiga toxin types and subtypes^4, 9^. Studies have shown that Stx2 to be involved more often than Stx1 and certain subtypes to be involved more often than others in causing human STEC infections, particularly in the development of serious illnesses^8, 10^. Enterohemorrhagic *E. coli* (EHEC), a term for a subset of STEC, is defined in part by the ability to produce attaching and effacing (A/E) lesions in the intestinal epithelium^11^. The A/E lesions, characterized by intimate bacterial attachment, cytoskeletal rearrangement and destruction of microvilli, require a pathogenicity island called the locus of enterocyte effacement (LEE), which encodes for a type III secretion system that injects bacterial effectors into host epithelial cells. Intimin, a 94- to 97-KDa outer membrane protein, encoded by the *eae* gene, mediates intimate attachment to enterocytes and is carried by all strains of EHEC^9^. Although a majority of STEC associated with severe illness (hemorrhagic colitis, HUS, hospitalization, and/or death) are positive for *eae*, many reports of *eae*-negative strains associated with severe illness exist because of alternative mechanisms of attachment. Furthermore, *eae*-positive strains are also associated with mild illness (only diarrhea)^9^.

Cattle are a major reservoir of STEC, which they harbor in the hindgut and then shed in the feces^12^. *Escherichia coli* O157 and six other non-O157 serogroups, O26, O45, O103, O111, O121, and O145, referred to as the ‘top-7’, are responsible for the majority of human STEC infections in North America^13–16^. The most common non-O157 serogroups associated with human STEC infections in the European Union include O26, O103, O91, O146, and O145^17, 18^. In recent years, non-O157 serotypes are increasingly recognized as causes of STEC infections in the USA. According to the Centers for Disease Control and Prevention estimation, 64% of STEC infections in the United States are caused by serogroups other than the O157^14^. In a summary of outbreaks of non-O157 STEC infections between 1990 and 2010 in the USA, 66% were caused by O111 or O26 and 84% were transmitted through food^17^. Additionally, 151 serogroups of *E. coli*, called ‘non-top-7 STEC’, have been shown to carry one or both Shiga toxin genes^16, 19–23^. At least 130 of the 151 STEC serogroups have been reported to be associated with human illnesses worldwide, often as sporadic infections and rarely as outbreaks^16, 19–22^. A number of studies have been reported on fecal shedding and prevalence of the top-7 STEC in cattle feces in the USA^24–28^. However, not much is known about fecal shedding and prevalence of the non-top-7 STEC serogroups in cattle feces. Also, prevalence of Shiga toxin types and subtypes of serogroups, which are shed in cattle feces but not commonly implicated in human STEC infections are not known. The information on the Shiga toxin types and subtypes are needed to assess their potential to cause human infection. In our previous studies conducted in 2013^26, 27^ and 2014^28^ that were designed to study the prevalence of the top-7 STEC, a number of *E. coli* strains (n = 351) positive for the *stx* gene but negative for the top-7 STEC serogroups were isolated. Our primary objectives in this study were to identify the serogroups of the 351 STEC strains isolated from cattle feces by multiplex PCR (mPCR) assays and conventional serological testing and determine subtypes of *stx* genes. Also, we developed and validated a novel mPCR assay of the six most common non-top-7 STEC O-groups (O2, O74, O109, O131, O168, and O171) to determine their prevalence in cattle feces collected from a commercial feedlot.

## Results

### Serogroups of non-top-7 STEC strains isolated from feedlot cattle feces

Of the 117 strains from the 2013 study, 94 (80.3%) were identified as belonging to 10 serogroups and 23 (19.7%) were negative for any of the 137 serogroups targeted by the 14 sets of mPCR assays (Table 1). Among the 10 identified serogroups, O168 (29.9%), O109 (20.5%), O171 (9.4%), and O74 (6.8%) accounted for 66.7% of the total. Of the 117 strains, 25 were EHEC pathotype and carried both *stx* and *eae* genes and the remaining 92 strains were categorized as STEC and were *eae* negative. Twenty-four (20.5%) of the 117 strains were positive for *stx*1, 91 strains (77.8%) were positive for *stx*2, and two strains (1.7%; serogroups O2 and O8) contained both. In the 2014 study, 197 (84.2%) of the 234 strains were identified as belonging to 14 serogroups and 37 (15.8%) were unidentified (Table 2). Among the 14 serogroups identified, five serogroups, O168 (30.8%), O109 (17.1%), O131 (12.0%), O2 (8.5%), and O104 (5.1%), accounted for 73.5% of the total isolates. Of the 234 strains, 43 (18.4%) were of the EHEC pathotype, and 191 (81.6%) were of STEC pathotype. Twenty-seven (11.5%) of the 234 strains carried *stx*1, 197 strains (84.2%) carried *stx*2 gene, and ten strains (4.3%) were positive for both (serogroups O2 [5], O8 [2], O113 [1], and O178 [2]).

**Table 1:** Detection of serogroups, virulence genes, and Shiga toxin gene subtypes in Shiga toxin-producing *Escherichia coli* strains (n = 117) isolated from feedlot cattle feces from one commercial feedlot by multiplex PCR assays (2013 study).

Serogroups	No of strains positive n = 117 (%)	Enterohemorrhagic *E. coli* (n = 25)			Shiga toxin-producing *E. coli* (n = 92)	
		*stx*1	*stx*2	*eae*	*stx*1	*stx*2
O131	1 (0.9)					1 *stx*2d
O160	1 (0.9)					1 *stx*2a
O169	1 (0.9)					1 *stx*2a
O8	3 (2.6)				1 *stx*1a	2 *stx*2a; 1 *stx*2d
O104	5 (4.3)				5 *stx*1c	
O2	5 (4.3)				1 *stx*1a	4 *stx*2a; 1 *stx*2c
O74	8 (6.8)	8 *stx*1a		8		
O171	11 (9.4)	1 *stx*1a		1		9 *stx*2a; 1 *stx*2c
O109	24 (20.5)		12 *stx*2a; 4 *stx*2c	16	8 *stx*1a	
O168	35 (29.9)				1 *stx*1a	31 *stx*2a; 1 *stx*2c; 2 *stx*2d
Unknown	23 (19.7)				1 *stx*1a	16 *stx*2a; 2 *stx*2c; 4 *stx*2d

**Table 2:** Detection of serogroups, virulence genes, and Shiga toxin gene subtypes in Shiga toxin-producing *Escherichia coli* strains (n = 234) isolated from cattle feces from eight commercial feedlots by multiplex PCR assays (2014 study).

Serogroups	No. of positive strains n = 234 (%)	Enterohemorrhagic *E. coli* (n = 43)			Shiga toxin-producing *E. coli* (n = 191)	
		*stx*1	*stx*2	*eae*	*stx*1	*stx*2
O76	1 (0.4)				1 *stx*1a	
O98	1 (0.4)	1 *stx*1a		1		
O113	1 (0.4)				1 *stx*1a	1 *stx*2a
O118	1 (0.4)	1 *stx*1a		1		
O178	2 (0.9)				2 *stx*1a	2 *stx*2a
O136	3 (1.3)					2 *stx*2d; 1 *stx*2a
O171	4 (1.7)					2 *stx*2a; 1 *stx*2c; 1 *stx*2d
O74	5 (2.1)	5 *stx*1a		5		
O8	7 (3.0)				4 *stx*1a	4 *stx*2a; 1 *stx*2d
O104	12 (5.1)				11 *stx*1c; 1 *stx*1a	
O2	20 (8.5)				7 *stx*1a	14 *stx*2a; 1 *stx*2c; 3 *stx*2d
O131	28 (12.0)	–	–	–		19 *stx*2a; 9 *stx*2c
O109	40 (17.1)		24 *stx*2a; 5 *stx*2c; 7 *stx*2d	36	3 *stx*1a	1 *stx*2a
O168	72 (30.8)					63 *stx*2a; 6 *stx*2d; 3 *stx*2c
Unknown	37 (15.8)					27 *stx*2a; 4 *stx*2c; 6 *stx*2d

Overall, among the 351 strains tested, the four dominant serogroups were O168 (n = 107; 30.5%), O109 (n = 64; 18.2%), O131 (n = 29; 8.3%), and O2 (n = 25; 7.1%). The distribution of the 16 groups in nine feedlots (1 feedlot in 2013 and 8 feedlots in 2014) is shown in Table 3. Of the four dominant serogroups, O168, O109, O131 and O2, all feedlots were positive for O168 and O109, and O131 was prevalent in seven and O2 was prevalent in six of the 9 feedlots (Table 3).

**Table 3:** Distribution of serogroups and number of strains of Shiga toxin-producing *Escherichia coli* isolated from feces of cattle collected from nine feedlots.

Feedlots	No. of isolates	Serogroups (no. of strains) identified
A	117	O168 (35), O109 (24), O171 (11), O74 (8), O2 (5), O104 (5), O8 (3), O131 (1), O160 (1), O169 (1), Unidentified (23)
B	25	O168 (9), O131 (5), O8 (1), O109 (1), Unidentified (9)
C	18	O168 (6), O109 (3), O131 (3), O76 (1), O2 (1), O8 (1), O74 (1), Unidentified (2)
D	28	O168 (9), O104 (5), O131 (4), O2 (2), O109 (2), O8 (1), Unidentified (5)
E	12	O131 (5), O168 (3), O109 (2), O171 (1), Unidentified (1)
F	34	O168 (18), O74 (4), O109 (4), O2 (3), O178 (1), Unidentified (4)
G	42	O109 (16), O131 (10), O168 (7), O8 (2), O171 (1), O98 (1), Unidentified (5)
H	44	O168 (11), O2 (8), O104 (7), O109 (5), O136 (3), O171 (2), O131 (1), Unidentified (7)
I	31	O168 (9), O109 (7), O2 (6), O8 (2), O113 (1), O118 (1), O178 (1), Unidentified (4)
Total	351	O168 (107), O109 (64), O131 (29), O2 (25), O104 (17), O171 (15), O74 (13), O8 (10), O136 (3), O178 (2), O169 (1), O160 (1), O118 (1), O113 (1), O98 (1), O76 (1), Unidentified (60)

### Comparison of serogrouping by serology and PCR

Serology identified 321 (91.5%) of the 351 strains as belonging to 16 serogroups and 30 (8.5%) strains were untypeable (Table 4). The identifications of the 260 strains of the following serogroups (no. of strains) by serology, O76 (1), O98 (1), O113 (1), O118 (1), O160 (1), O169 (1), O178 (2), O136 (3), O8 (10), O2 (24) O74 (13), O171 (15), O104 (17), O109 (63) and O168 (107) matched the identification by PCR method. The serogroups identified by PCR, but not by serology (no. of strains), included O2 (1), O109 (1), and O131 (29). One of the 25 strains of O2 based on PCR was identified as O8 by serology. The 29 O131 strains identified by PCR were positive for O2 (1), O152 (1), O156 (26) by serology and one strain was untypeable. Among the 64 O109 strains, 63 were identified as O109 and one strain was identified O156. Of the 60 unidentified strains by PCR, serology indicated 29 were untypeable, one was O11, and the remaining 30 were identified as O152. In instances when serology identified serogroups differently (O2, O8, O11, O152, and O156) or were untypeable, PCR assays with primers targeting those serogroups described by Iguchi *et al*^38^ and DebRoy *et al*^31, 37^ were also negative.

**Table 4:** Comparison of serogroup identification by PCR and serology of Shiga toxin gene-positive *Escherichia coli* strains (n = 351) isolated from feedlot cattle feces.

No. of strains	Serogrouping by PCR (no. of strains)	Serogrouping by serology (no. of strains)
1	O76 (1)	O76 (1)
1	O98 (1)	O98 (1)
1	O113 (1)	O113 (1)
1	O118 (1)	O118 (1)
1	O160 (1)	O160 (1)
1	O169 (1)	O169 (1)
2	O178 (2)	O178 (2)
3	O136 (3)	O136 (3)
10	O8 (10)	O8 (10)
13	O74 (13)	O74 (13)
15	O171 (15)	O171 (15)
17	O104 (17)	O104 (17)
25	O2 (25)	O2 (24), O8 (1)^a^
29	O131 (29)	O2 (1), O152 (1), O156 (26)^b^, untypeable (1)
64	O109 (64)	O109 (63), O156 (1)^c^
107	O168 (107)	O168 (107)
60	Unidentified (60)	O152 (30), O11 (1)^d^, untypeable (29),

^a^O8 strain was positive for O2 by PCR with primers from Iguchi et al.^38^ and DebRoy et al.^29, 37^.

^b^Strains O2 (1), O152 (1), and O156 (26) were negative by PCR for O2, O152 and O156 with primers from Iguchi et al.^38^ and DebRoy et al.^29, 37^.

^c^Strain O156 (1) was positive for O109 by PCR with primers for O109 from Iguchi et al.^38^ and DebRoy et al.^29, 37^.

^d^Strains O152 (30) and O11 (1) were negative by PCR for O152 and O11 with primers from DebRoy et al.^29, 37^.

### Shiga toxin gene subtypes

**Figure 1:**
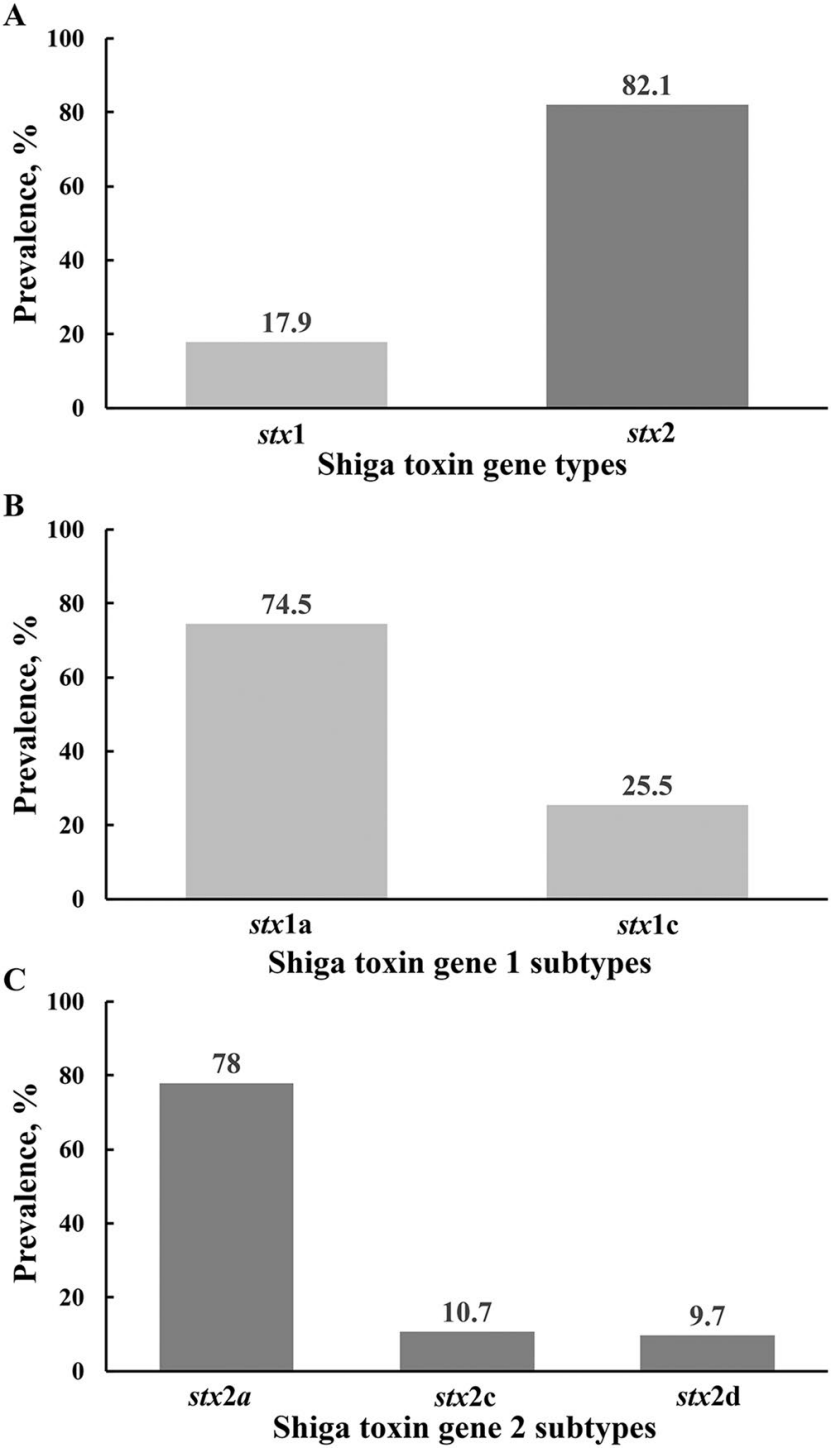
Prevalence of Shiga toxin types (**A**), Shiga toxin subtypes (**B**) and Shiga toxin 2 subtypes (**C**) among the non-top-7 Shiga toxin-carrying *Escherichia coli* strains (n = 35) isolated from feedlot cattle feces.

Of the total 351 strains (2013 and 2014 studies), 63 (18.0%) had *stx*1 gene and 300 (85.5%) had *stx*2, which included 12 strains positive for both (Fig. 1A). The majority of the *stx*1 was of *stx*1a subtype (47/63; 74.5%) and the other 16 strains (16/63; 25.5%) possessed *stx*1c subtype (Fig. 1B). The majority of *stx*2 was of the *stx*2a subtype (234/300; 78%) and the remaining were *stx*2c (32/300; 10.7%) and *stx*2d (34/300; 9.7%) (Fig. 1C).

### Validation of the mPCR assay to detect O2, O74, O109, O131, O168, and O171 serogroups in cattle feces

**Figure 2:**
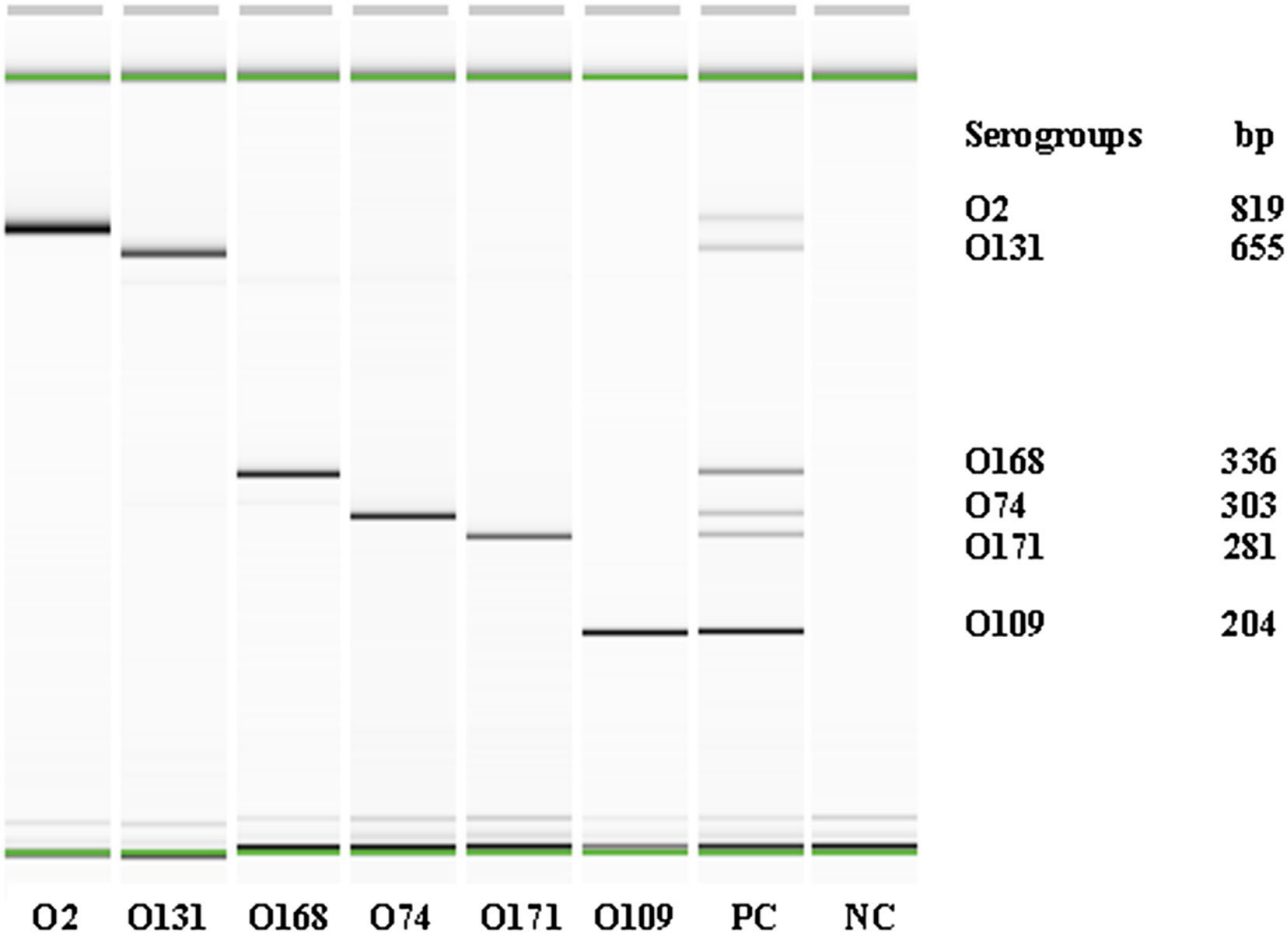
QIAxcel image of the amplicons of serogroup-specific genes of six individual and pooled Shiga toxin-producing *Escherichia coli* amplified by six-plex PCR assay (PC = Positive control [mixture of all 6 serogroups]); NC = Negative control).

The specificity of each primer pair was tested individually using the DNA from the pooled strains of the six serogroups targeted in the assay. The primers amplified the targeted serogroup only (Fig. 2). None of the non-targeted strains of the top-7 (7 strains) and of the non-top-7 STEC serogroups (132 strains belonging to 132 serogroups) yielded any amplification (data not shown). The initial bacterial concentrations of the six STEC strains used for spiking feces were 3.2 × 10^8^ CFU/ml for O2, 5.6 × 10^8^ CFU/ml for O74, 3.1 × 10^8^ CFU/ml for O109, 4.1 × 10^8^ CFU/ml for O131, 4.5 × 10^8^ CFU/ml for O168, and 6.5 × 10^8^ CFU/ml for O171. The detection limit of the assay for spiked fecal samples was ~ 10^6^ CFU/ml and ~ 10^2^ CFU/ml before and after enrichment in EC broth, respectively (data not shown).

### Prevalence of O2, O74, O109, O131, O168, and O171 serogroups in feedlot cattle feces

**Figure 3:**
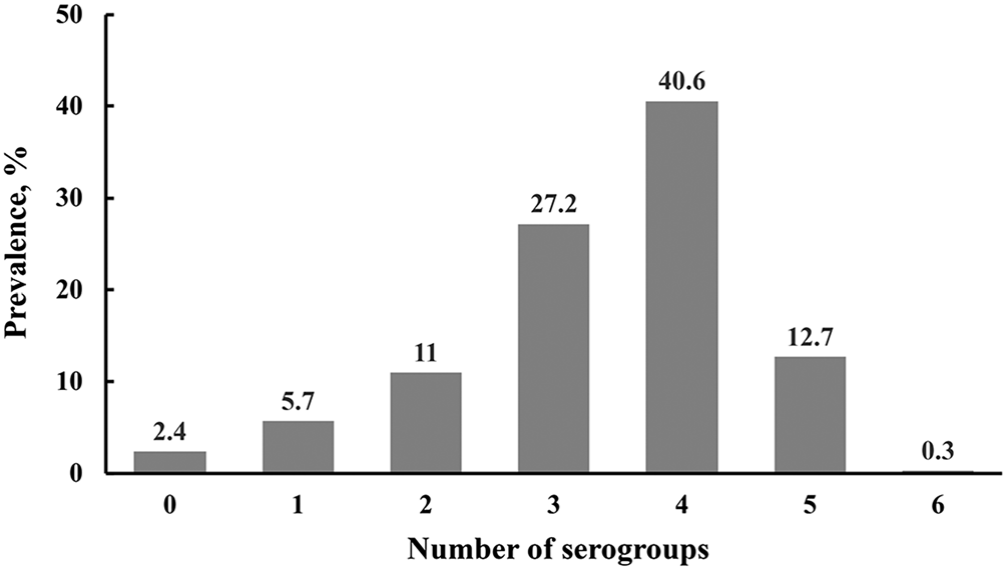
Percentage of fecal samples that tested positive for none, one or more of the six O serogroups (O2, O74, O109, O131, O168 and O174) of the predominant non-top-7 Shiga toxin gene-positive *Escherichia coli* in feedlot cattle.

A total of 576 fecal samples collected from cattle in a commercial feedlot were subjected to the mPCR assay. Model-adjusted mean prevalence and 95% confidence intervals of test positive samples for O serogroups and virulence genes are shown in Table 5. Of the six serogroups, O109 (91.6%), O171 (87.5%), O168 (79.5%), and O2 (59.5%) were the most predominant. A high proportion of the samples positive for the serogroups tested positive for the three virulence genes, *stx*1, *stx*2 and *eae*. The majority of the fecal samples (80.5%) tested positive for 3 to 5 serogroups of the six non-top-7 STEC (Fig. 3).

**Table 5:** Model-adjusted mean prevalence and 95% confidence intervals (CI) of *Escherichia coli* O serogroups and virulence genes that encode for Shiga toxin 1 (*stx*1) and 2 (*stx*2) and intimin (*eae*) determined by multiplex PCR in fecal samples (n = 576) from feedlot cattle.

Serogroups	Mean prevalence, % (95% CI)					
	O group	O group + *stx*1	O group + *stx*2	O group + *eae*	O group + *stx*1 and or *stx*2	O group + *stx*1 and or *stx*2 and *eae*
O2	59.5 (50.6–67.8)	42.1 (34.2–50.4)	57.1 (48.7–65.1)	57.9 (49.6–65.7)	58.0 (49.3–66.3)	56.7 (48.7–64.4)
O74	17.4 (10.6–27.4)	12.7 (7.5–20.7)	16.5 (10.1–25.8)	17.1 (10.3–26.9)	16.8 (10.2–26.4)	16.4 (9.8–26.0)
O109	91.6 (86.8–94.7)	60.8 (52.9–68.1)	87.6 (82.2–91.5)	89.1 (83.6–92.9)	88.7 (83.6–92.4)	86.9 (81.7–90.8)
O131	0.9 (0.2–3.8)	0.7 (0.2–2.5)	0.9 (0.2–3.8)	0.9 (0.2–3.8)	0.9 (0.2–3.8)	0.9 (0.2–3.8)
O168	79.5 (70.9–86.0)	54.0 (44.1–63.7)	76.9 (68.7–83.5)	77.8 (69.4–84.5)	77.9 (69.5–84.5)	76.3 (68.0–83.0)
O171	87.5 (81.1–91.9)	58.1 (49.8–66.0)	83.4 (77.7–87.9)	85.7 (79.0–90.6)	84.7 (78.6–89.3)	83.3 (76.9–88.2)

## Discussion

Serogrouping of *E. coli* is based on the chemical composition of the O antigen of the lipopolysaccharide located in the outer membrane of the cell envelope^30, 31^. A total of 187 serogroups of *E. coli* have been identified^29^. Shiga toxin-producing *E. coli* (STEC) constitute a major pathotype and includes as many as 158 serogroups^16, 19–23^. A number of studies have reported on the isolation and prevalence of the O157 and the top-6 non-O157 serogroups in cattle feces and other sample matrices by culture method^24, 26, 32–36^. Serogroup identification by agglutination reactions carried out in microtiter plates with a panel of antisera generated by immunization of rabbits with different O group reference strains is a simple and traditional method^37^. However, the method is subjective, and because of the cost of generating and storing antisera, only a few reference laboratories are capable of routinely carrying out these tests. Furthermore, almost always a small proportion of the *E. coli* isolates are serologically untypeable because of no agglutination, autoagglutination, or cross-reactions^37^. Therefore, there is a shift away from serological phenotypic testing towards molecular genotyping based on genes required for biosynthesis and export of O antigens^29, 38^. Because of specificity, ease, and ability to multiplex, PCR assays have become the method of choice to identify serogroups of *E. coli*. A number of conventional and real-time mPCR assays have been developed to identify serogroups that are more relevant to clinical infections^39–48^. The serogroups included top-7 STEC (in the US) and a few other minor serogroups, such as O5, O15, O55, O76, O91, O113, O118, O123, O128, O146, O165, O172, and O177, which were isolated from human clinical infections.

In contrast, PCR assays targeting the minor non-top-7 STEC are limited. Iguchi et al. designed primer pairs to develop 20 mPCR assays, with each assay targeting six to nine serogroups, to detect 147 of the 187 serogroups that included STEC and non-STEC^38^. DebRoy et al. have described individual primer pairs and PCR assays for 185 of the 187 serogroups^29^. Two serogroups, O14 and O57, were not included because neither contain O-antigen biosynthesis gene clusters^49, 50^. We have developed and validated 14 sets of mPCR assays, each targeting seven to 12 serogroups, to detect 137 STEC serogroups that have been detected in cattle feces^23^.

Using the 14 sets of mPCR assays, serogroups of 291 strains out of 351 (82.9%) were identified as belonging to 16 serogroups, and the remaining 60 (17.1%) were unidentified. However, of the 60 strains unidentified by our PCR, serology identified 31 strains as O11 (1) and O152 (30) and the remaining 29 as untypeable. The strains that were identified differently by serology (O2, O11, O131, O109, O152, and O156) also tested negative by PCR with primers described by DebRoy *et al*^37^ and Iguchi *et al*^38^. The discrepancy between PCR and serology has been reported previously^50^. In certain serogroups with similar nucleotide sequences, serology may not show any cross reactivity, which could be due to posttranslational modifications of the proteins resulting in changed epitopes in antigens^50, 51^. In silico serogrouping based on assembled whole genome sequence (WGS)^51^ or raw short read WGS data^52^ may identify the serogroup and likely provide reasons as to why PCR was not able to identify them.

Based on both methods of serogrouping, only 19 STEC serogroups were identified; 16 by PCR and 3 additional serogroups by serology. It is important to recognize that although as many 151 non-top-7 serogroups of STEC have been isolated from cattle feces, only 19 STEC serogroups were obtained from fecal samples collected from 9 feedlots. Because the isolates were obtained from immunomagnetic beads (IMS) specific for the top-7 STEC, the serogroups may not represent the true distribution of STEC serogroups in cattle feces.

Interestingly, the majority of the non-top-7 STEC strains possessed *stx*2 (300/351; 85.5%), which is in contrast to what has been observed for the six serogroups of non-O157 STEC, which primarily possess *stx*1^13, 34, 53, 54^. The predominance of *stx*2 suggests the potential risk of the non-top-7 STEC to cause human infections. Shiga toxin 2 was about 400 times more toxic in a mouse infection model^5^ and was more commonly associated with complications of human STEC illnesses than Shiga toxin 1^4, 55, 56^. Shiga toxin 2, particularly in association with intimin*,* results in a higher risk for severe infections^57^, although Shiga toxin 2 without intimin can cause severe infection as evidenced in the O104:H4 outbreak in Germany in 2011^58^. The EHEC pathotype, a subset of STEC, was once considered to be associated more often with severe STEC infections. In a scientific opinion agreed upon by the European Food Safety Authority Panel, the EHEC terminology is considered obsolete and the recommendation was to use STEC for all *stx-*positive strains^8^.

The predominance of the subtypes *stx*1a and *stx*2a in the non-top-7 STEC identified in this study is similar to previous reports of their dominance among O157 and top-6 non-O157 strains of human clinical origin^56, 59, 60^. The *stx*1a is often produced by LEE-positive strains of STEC and have the potential to cause severe infections^13^. Epidemiological data from human infections indicate a stronger association of *stx*2a- and *stx*2d- positive strains with severe hemorrhagic enteritis, including HUS^56, 61, 62^. These two subtypes were more cytotoxic than *stx*2b and *stx*2c in an in vitro potency assay^63^.

Of the total 16 serogroups identified in the study, seven serogroups, O2, O74, O104, O109, O131, O168, and O171, accounted for 76.9% (270/351) and 92.8% (270/291) of the total and serogroup-identified strains, respectively. Because the 351 strains used in the study were from immunomagnetic beads that targeted the six non-O157 serogroups, the dominance of a few serogroups in isolated strains is not indicative of their prevalence in cattle feces. Also, IMS beads are not available for these serogroups, which rule out culture method to selectively isolate, identify and determine their prevalence. Therefore, a novel mPCR assay of the dominant serogroups was designed, validated, and utilized to determine the prevalence in feces of feedlot cattle. The PCR assay did not include O104 because we had previously developed a mPCR assay for the top-7 STEC and O104^64^ and determined the prevalence of O104 serogroups and characterized the isolated serotypes in cattle feces^65, 66^. The reason for including O104 with the top-7 STEC at the time was because O104:H4, a hybrid pathotype of STEC and enteroaggregative *E. coli,* was involved in a major foodborne outbreak in Germany in 2011^67^. Of the six serogroups, prevalence of four serogroups, O2 (59.2%), O109 (91%), O171 (86.5%), and O168 (78.1%), were higher than the other two serogroups. It should be noted that this is only a preliminary finding, based on PCR assay from fecal samples collected from one feedlot and additional studies, possibly including culture methods, are needed. In the majority of the fecal samples (80.8%), multiple serogroups (three or more) were present, likely because of the high prevalence of three serogroups (> 78% of O168, O171, and O109). This is in contrast to the prevalence of the six major non-O157 serogroups, in which the majority of the samples (68.1%) were positive for one or two serogroups^26^. The four serogroups, O2, O109, O171 and O168, have been frequently isolated from feces of healthy cattle^68–71^. However, this is the first study that provides prevalence estimates of these six groups in feces from commercial feedlot cattle (with natural shedding).

At least 130 of the 151 serogroups of non-top-7 STEC have been reported to be associated with clinical cases of diarrhea, and a few serogroups and serotypes have been associated with severe forms of infections, including complication of HUS^16, 20, 22, 34, 72–78^. Certain serogroups, such as O2, O8, and O113, and specifically certain serotypes within these serogroups, have been reported to cause outbreaks associated with consumption of contaminated beef in the US, European countries, and Australia^20, 77, 79^. Serogroup O113 (mostly the H21 serotype) has been associated with severe cases of hemorrhagic colitis and HUS in the US and other countries^79–81^.

In conclusion, cattle harbor and shed in feces a number of serogroups of STEC other than the top-7 responsible for the majority of foodborne STEC infections. The majority of the non-top-7 strains isolated and serogrouped possessed *stx*2 and were of the subtype *stx*2a, suggesting their potential to cause severe infections in humans. Although a majority of the non-top-7 STEC have been shown to cause sporadic infections, a few serogroups, notably O2, O8, O91, and O113 have been implicated in outbreaks and serious infections. The fecal prevalence of a few serogroups, namely O2, O109, O168, and O171, was high in feedlot cattle. The importance of these non-top-7 STEC as foodborne pathogens in humans is not known. Not much is known about the prevalence of these STEC serogroups on cattle hides and carcass surfaces and in beef products and other food matrices in the USA, largely because detection strategies have not been developed and validated. Our study provides information on the detection and prevalence of major serogroups of non-top-7 STEC in cattle.

## Materials and methods

### Identification of serogroups of non-top-7 STEC strains isolated from feedlot cattle feces

A total of 351 *stx-*positive isolates that were negative for the top-7 STEC serogroups (O26, O45, O103, O111, O121, O145, and O157), based on mPCR assay^39^, obtained from two feedlot studies conducted in 2013 (n = 117 isolates^26, 27^) and 2014 (n = 234 isolates^28^) were used. In the 2013 and 2014 studies, the culture method to detect and isolate six serogroups of non-O157 STEC involved enrichment, serogroup-specific IMS, plating on modified Possé (MP) medium and serogroup and virulence genes confirmation of putative isolates by PCR^26^. Because the chromogenic colonies of the six non-O157 serogroups on MP medium were phenotypically indistinguishable, a pool of 10 randomly picked chromogenic colonies from the plate inoculated with IMS beads was prepared and tested by mPCR assay targeting seven serogroups of STEC^45^. If positive for any of the seven serogroups, then each of the 10 colonies was tested individually by a mPCR assay targeting the seven serogroups (O26, O45, O103, O111, O121, O145, and O157) and three virulence genes (*stx*1, *stx*2, and *eae*)^26, 39^. Isolates positive for *stx*1 and/or *stx*2 and negative for any of the seven serogroups were considered as STEC other than the top-7 (non-top-7). The 117 isolates from the 2013 study were from a total of 576 fecal samples collected from 24 pens in a single commercial feedlot. The 234 isolates from the 2014 study were from a total of 1,886 fecal samples collected from 64 pens in eight commercial feedlots located in two major U. S. beef cattle states. The isolates stored in cryobeads (CryoCare, Key Scientific Products, Round Rock, TX) at -80° C were streaked onto blood agar plates (BAP; Remel, Lenexa, KS) and incubated overnight at 37° C. A single colony of each strain was suspended in 50 µl distilled water, boiled for 10 min, centrifuged, and the supernatant was used as the template in the 14 sets of mPCR assays designed to detect 137 serogroups of non-top-7 STEC^23^. The serological tests for O-group determination, based on agglutination^31^, were conducted at the *E. coli* Reference Center (Pennsylvania State University).

### Subtyping of *stx* genes

The subtypes of *stx*1 and *stx*2 genes of the 351 STEC strains were determined according to the protocol described by Shridhar *et al*^54^. Briefly, a colony from BAP was suspended in distilled water, boiled, centrifuged and the lysate was used to amplify *stx*1 and *stx*2 genes by touchdown PCR. PCR products were purified using a QIAquick PCR purification kit (Qiagen, Valencia, CA) and shipped to Genewiz, Inc., (South Plainfield, NJ) for nucleotide sequencing. The chromatogram data of each sequence was individually analyzed for conflicts and secondary peaks, and consensus sequences were produced using the CLC Main Workbench software (Qiagen, Valencia, CA). The nucleotide sequences were conceptually translated to amino acid sequences and Shiga toxin subtypes were determined based on the amino acid motifs that define each *stx* subtype^7^.

### Development and validation of a mPCR assay targeting O2, O74, O109, O131, O168, and O171 serogroups.

#### Primers design

The serogroup-specific *wzx* gene, which encodes for the transmembrane lipid transporter enzyme or flippase, required for the O-polysaccharide export, was targeted in this assay. The primers were designed based on the nucleotide sequences of the target gene for each of the six serogroups obtained from the GenBank database. The sequences for each serogroup were aligned using ClustalX version 2.0^82^, and the primers that amplify the targets with distinct amplicon sizes that can be differentiated by capillary gel electrophoresis were chosen for the study.

#### Assay running conditions

The reaction consisted of 10 μl of BioRad iQ multiplex powermix, 1 µl of six pairs of primer mix (8 pM/µl for each primer), 2 µl of template, and 7 µl of water (total reaction volume = 20 µl). The PCR running conditions consisted of an initial denaturation at 94^°^ C for 5 min followed by 30 cycles of denaturation at 94^°^ C for 30 s, annealing at 72^°^ C for 30 s, extension at 68^°^ C for 75 s, and a final extension step at 68^°^ C for 7 min. The primer sequences and amplicon sizes are provided in Table 6.

**Table 6:** Target genes, primer sequences, and amplicon sizes of the six-plex PCR assay.

Genes	Primers	Sequence	Amplicon size (bp)
*wzx*_O2/O50_	Forward	TGGCCTTGTTCGATATACTGCGGA	819
	Reverse	TCACGAGCTGAGCGAAACTGTTCA	
*wzx*_O131_	Forward	GTGATTTCTGGGGCAACATT	655
	Reverse	AAGCCTGCCCTAAACAAAGC	
*wzx*_O168_	Forward	TGTCGACTTTGGGAAATGTGG	336
	Reverse	CTGCAGAGGCCAATTCAGGT	
*wzx*_O74_	Forward	CTGGTCAATGGCAAGCTGTA	303
	Reverse	ATGCAAAAATCCAAGCCAAT	
*wzx*_O171_	Forward	TGCTCAAGTGGCATGCAGAT	281
	Reverse	TGCAACCTGATATCCAGCAGT	
*wzx*_O109_	Forward	TCTCTCTCGACATACCCGCGCTT	204
	Reverse	ACCGTAGCCCAAAGAGCCACA	

#### Specificity of the six-plex PCR assay

A collection of 139 strains belonging to top-7 serogroups (O26, O45, O103, O111, O121, O145, and O157) and non-top-7 serogroups (O1, O3, O4. O5, O6, O7, O8, O9, O10, O11, O12, O13, O15, O16, O17, O18, O19, O20, O21, O22, O23, O25, O27, O28, O29, O32, O33, O35, O37, O38, O39, O40, O41, O43, O46, O48, O49, O51, O53, O54, O55, O56, O58, O60, O62, O63, O64, O65, O66, O69, O70, O71, O75, O76, O78, O79, O80, O81, O82, O83, O84, O85, O86, O87, O88, O89, O90, O91, O92, O93, O96, O98, , O100, O102, O104, O105, O107, O108, O110, O112, O113, O114, O115, O116, O118, O119, O120, O123, O124, O125, O126, O128, O130, , O132, O133, O136, O138, O139, O140, O141, O142, O143, O144, O146, O147, O148, O149, O150, O152, O153, O154, O156, O159, O160, O161, O163, O165, O166, O167, O169, O170, , O172, O173, O174, O175, O176, O177, O178, O179, O180, O181, and O182) were used to determine the specificity of the assay. The strains were from our culture collection, *E. coli* Reference Center at Pennsylvania State University, Michigan State University, University of Nebraska, and Food and Drug Administration^23^. One strain of each serogroup was used. The strains stored at -80 °C were grown on BAP to obtain single colonies. One or two colonies were suspended in 1 ml of water, boiled and centrifuged as before and the lysate was used in mPCR assay.

#### Sensitivity of the six-plex mPCR assay with cattle fecal samples spiked with pure cultures

Ten pen-floor fecal samples, collected from feedlot cattle housed at the Beef Cattle Research Center, Kansas State University, were tested by the six-plex PCR assay targeting O2, O74, O109, O131, O168, and O171 serogroups. A fecal sample that tested negative for the six serogroups was selected for spiking with pure cultures. Six STEC strains, 14.1652 (O2), 4558-1 (O74), 12662-2 (O109), 12.3205 (O131), 15.0133 (O168), and 1044-1 (O171), previously isolated from cattle feces, were used to spike fecal samples. Three strains (4558-1, 12662-2 and 1044-1) were from our culture collection^83^ and the remaining three were from the *E. coli* Reference Center. Each strain, grown individually in Luria–Bertani (LB) broth, was serially diluted ten-fold (10^–1^ to 10^–8^) and 100 µl of 10^–5^, 10^–6^ and 10^–7^ dilutions were spread-plated onto BAP (four plates per dilution) to determine the initial bacterial concentrations (CFU/ml). Approximately 50 g of fecal sample selected for spiking was suspended in 450 ml of *E. coli* (EC) broth (Difco, Becton, Dickinson Co., Sparks, MD) and dispensed into sterile tubes (9.4 ml/tube). Serial dilutions (10^–0^ to 10^–8^) of pure cultures of each of the six serogroups were inoculated (100 µl) into a 9.4 ml fecal suspension. Spiked fecal samples were vortexed and incubated at 40 °C for 6 h. One ml of pre- and post-enrichment spiked fecal suspensions were boiled for 10 min and centrifuged at 9,300 × g for 5 min. DNA cleanup of pre- and post-enrichment fecal suspensions was performed using the GeneClean Turbo Kit (MP Biomedicals LLC, Solon, OH), and subjected to the six-plex PCR assay. The experiment was repeated with a different fecal sample that was tested negative for the serogroups.

#### Applicability of the six-plex PCR assay to detect O2, O74, O109, O131, O168, and O171 serogroups in cattle feces

Fecal samples collected from a commercial feedlot in the 2013 study to determine the prevalence of top-7 STEC serogroups^26, 27^ were used. Fecal samples were collected weekly for 12 weeks in the summer (June–August). Each week, 24 pen-floor fecal samples were collected from each of two pens of finishing cattle a day before transport of cattle for slaughter. A total of 576 fecal samples from 24 pens were collected. Fecal samples were enriched in *E. coli* broth^45^ by incubating at 40 °C for 6 h and stored at − 80 °C. Enriched fecal samples were thawed and the DNA extracted and purified (as described above) was used as the template for the six-plex PCR assay.

### Statistical analysis

Descriptive statistics (frequency tables [number and %]) were computed to describe the cumulative fecal prevalence for serogroup, STEC, and EHEC O groups. A sample was considered serogroup positive, if it tested positive for the serogroup only (disregarding presence or absence of virulence genes). Model-adjusted cumulative sample-level prevalence estimates and their 95% confidence intervals of test positive samples for O serogroups and virulence genes were estimated from model intercepts using generalized linear mixed models. Outcomes consisted of: 1) sample-level serogroup prevalence of O2, O74, O109, O131, O168, and O171 groups, 2) sample-level STEC prevalence of O2, O74, O109, O131, O168, and O171 (samples positive for serogroup and *stx*1 and or *stx*2), and 3) sample-level EHEC prevalence of O2, O74, O109, O131, O168, and O171 (samples positive for serogroup and *stx*1 and or *stx*2 and *eae*). Statistical models were fitted in Proc Glimmix (SAS 9.4; SAS Institute Inc., Cary, NC) using a binary distribution, logit link, residual pseudo-likelihood estimation, Kenward-Rogers degrees of freedom approximation, and random intercepts for pen and week to account for the clustering of pens nested within sampling week.
